# Diagnostic performance of ELISA, IFAT and Western blot for the detection of anti-*Leishmania infantum* antibodies in cats using a Bayesian analysis without a gold standard

**DOI:** 10.1186/s13071-017-2046-3

**Published:** 2017-03-13

**Authors:** Maria Flaminia Persichetti, Laia Solano-Gallego, Angela Vullo, Marisa Masucci, Pierre Marty, Pascal Delaunay, Fabrizio Vitale, Maria Grazia Pennisi

**Affiliations:** 1 Istituto Zooprofilattico Sperimentale della Sicilia, A. Mirri, Via G. Marinuzzi 3, Palermo, 90129 Italy; 2 Departament de Medicina i Cirurgia Animals. Facultat de Veterinària, Universitat Autònoma de Barcelona, Bellaterra, Cerdanyola, 08193 Barcelona Spain; 3 Dipartimento di Scienze Veterinarie, Università degli Studi di Messina , Polo Universitario Annunziata, Messina, 98168 Italy; 4 Centre Hospitalier Universitaire de Nice, Faculté de Médecine, Université de Nice-Sophia Antipolis, Inserm U 1065, Hôpital de l’Archet, 151, route de Saint Antoine de Ginestière, CS 23079 06202 Nice Cedex 3, France; 5 Parasitologie-Mycologie, Hôpital de l’Archet, Centre Hospitalier Universitaire de Nice, France-MIVEGEC, UMR IRD224 - CNRS 5290 - Université de Montpellier, Montpellier Cedex 5, France; 6 Centro di Referenza Nazionale per le Leishmaniosi (C.Re.Na.L), Istituto Zooprofilattico Sperimentale della Sicilia, A. Mirri, Via G. Marinuzzi 3, Palermo, 90129 Italy

**Keywords:** Bayesian analysis, Cat, Diagnostic performance, ELISA, Gold standard, IFAT, *Leishmania*, Leishmaniosis, Serological diagnosis, Western blot

## Abstract

**Background:**

Anti-*Leishmania* antibodies are increasingly investigated in cats for epidemiological studies or for the diagnosis of clinical feline leishmaniosis. The immunofluorescent antibody test (IFAT), the enzyme-linked immunosorbent assay (ELISA) and western blot (WB) are the serological tests more frequently used. The aim of the present study was to assess diagnostic performance of IFAT, ELISA and WB to detect anti-*L. infantum* antibodies in feline serum samples obtained from endemic (*n* = 76) and non-endemic (*n* = 64) areas and from cats affected by feline leishmaniosis (*n* = 21) by a Bayesian approach without a gold standard.

**Methods:**

Cut-offs were set at 80 titre for IFAT and 40 ELISA units for ELISA. WB was considered positive in presence of at least a 18 KDa band. Statistical analysis was performed through a written routine with MATLAB software in the Bayesian framework. The latent data and observations from the joint posterior were simulated in the Bayesian approach by an iterative Markov Chain Monte Carlo technique using the Gibbs sampler for estimating sensitivity and specificity of the three tests.

**Results:**

The median seroprevalence in the sample used for evaluating the performance of tests was estimated at 0.27 [credible interval (CI) = 0.20–0.34]. The median sensitivity of the three different methods was 0.97 (CI: 0.86–1.00), 0.75 (CI: 0.61–0.87) and 0.70 (CI: 0.56–0.83) for WB, IFAT and ELISA, respectively. Median specificity reached 0.99 (CI: 0.96–1.00) with WB, 0.97 (CI: 0.93–0.99) with IFAT and 0.98 (CI: 0.94–1.00) with ELISA. IFAT was more sensitive than ELISA (75 *vs* 70%) for the detection of subclinical infection while ELISA was better for diagnosing clinical leishmaniosis when compared with IFAT (98 *vs* 97%).

**Conclusions:**

The overall performance of all serological techniques was good and the most accurate test for anti-*Leishmania* antibody detection in feline serum samples was WB.

## Background

Leishmaniosis due to *Leishmania infantum* is a zoonotic vector-borne disease of worldwide importance, transmitted by phlebotominae sand flies. Dogs are the primary reservoir host; however other animal species can be infected, including cats [1, 2]. The role of cats as reservoirs of *L. infantum* is strongly suspected as infected cats are able to transmit the parasite to vector sand flies [3]. Moreover, clinical cases of feline leishmaniosis and subclinical infections due to *L. infantum* are increasingly being reported in Europe [1, 2].

IFAT and ELISA are amongst the most common serological techniques used for the diagnosis and for clinical and research studies on canine and feline *L. infantum* infection [1, 4–6]. For both IFAT and ELISA, quantification using antibody titer or optical density allows classification of antibody levels against *L. infantum* antigens. IFAT method is considered the reference technique by the World Organization for Animal Health (OIE) [7]. However, this technique depends on the operator’s skills and experience for the microscopical reading of IFAT antigen slides [4, 8]. Moreover, appropriate setting of cut-off level is crucial in determining sensitivity (Se) and specificity (Sp) of this test. Conversely, reading of ELISA plates is rapidly operated in a plate reader at the required absorbance and, in addition to the selected cut-off, Sp and Se strongly depend on the kind of antigen used [9, 10]. Western blot (WB) analysis, mainly a qualitative serological method, distinguishes the molecular weight of the *L. infantum* antigens stimulating antibody production, but is less frequently used in veterinary practice for the diagnosis of leishmaniosis [11]. One potential field of application of WB method is the discrimination between subclinical infections and disease [12].

Numerous epidemiological studies demonstrated the presence of anti-*Leishmania* antibodies in feline sera by means of different techniques such as IFAT, ELISA or WB as previously reviewed elsewhere [1, 2]. It is important to highlight that sensitivity and specificity estimates of these serological methods in cats unfortunately were rarely evaluated [4, 11]. However, ELISA and WB tests were reported to be more sensitive than IFAT [10, 13–15]. Variation in sensitivity and specificity is mainly attributable to differences among the reference population studied and sampling strategies that are used for the validation procedure [16]. In addition, the serological diagnostic techniques used may have considerable influence on the estimate obtained for the true seroprevalence; however, comparative studies on serological techniques used in cats are limited and scarce [4, 11, 17].

True differences of test accuracy among studies are not directly observable because studies are not free of random and systematic errors such as technical variation of test characteristics (among laboratories; by time), laboratory proficiency, choice of gold standard or cut-off value for interpretation, and handling of intermediate or uninterpretable results [16].

A common practice in many diagnostic accuracy studies is to evaluate a novel test by using another test as a gold standard. This approach yields strongly biased test accuracy estimates if the test considered gold standard have Se and Sp not approaching 100%. This may occur with leishmaniosis caused by *L. infantum* as a gold standard technique does not exist for diagnosis of infection or disease [18]. In order to avoid imperfect standard bias, we used the Bayesian method which has been proposed to estimate accuracy parameters of the tests [19, 20] by an iterative Markov Chain Monte Carlo (MCMC) technique using the Gibbs sampler for estimating Se and Sp.

The aim of the present study was to assess diagnostic performance of IFAT, ELISA and WB to detect anti-*L. infantum* antibodies in feline serum samples obtained from endemic (*n* = 76) and non-endemic (*n* = 64) areas and from cats affected by feline leishmaniosis (*n* = 21) by a Bayesian approach without a gold standard.

## Methods

### Feline serum samples

Overall, 161 residual feline sera samples were obtained in 2013 as described below and stored at−20 °C until analyzed.

#### Feline sera from a non-endemic area of leishmaniosis

Sixty-four feline serum samples of cats seen for medical reasons at Beamount and Queen Mother Hospitals from Royal Veterinary College University of London (UK), where leishmaniosis is not endemic, were obtained. No travel history, clinical or clinicopathological information was available for these cats.

#### Feline sera of cats living in an endemic area of leishmaniosis

Seventy-six sera were from adult cats living in the South of Italy and exposed to at least one sand fly transmission season. They were collected at three veterinary clinics from cats admitted for elective surgery (*n* = 35) or annual health check (*n* = 4) and from 37 sick cats. One or more of the following clinical conditions were detected in these sick cats: anemia (*n* = 23), otitis (*n* = 11), skin disease (*n* = 10), lower urinary tract disease (*n* = 9), stomatitis (*n* = 7), ocular disease (*n* = 5), fever (*n* = 3), respiratory disease (*n* = 3). Two veterinary clinics were located in the province of Messina (Ospedale Didattico Veterinario, Università degli Studi di Messina, Messina and Ambulatorio Veterinario S. Lucia, Lipari) and one in Reggio Calabria (Clinica Veterinaria Camagna).

#### Feline sera of cats affected by feline leishmaniosis

Twenty-one sera were of cats from South of Italy with clinical and clinicopathological findings compatible with feline leishmaniosis and diagnosis confirmed by at least two different parasitological methods among cytology, immunohistochemical staining, PCR, culture and xenodiagnosis [1]. Clinical findings included lymph node enlargement, skin and mucosal lesions (nodules, ulcers, crusts), weight loss, chronic stomatitis, ocular lesions; clinicopathological abnormalities found were normocytic normochromic anemia, leukopenia, thrombocytopenia, pancytopenia, hyperproteinemia, hypoalbuminemia, hypergammaglobulinemia, azotemia and increased urinary protein/creatinine ratio.

### Serological techniques

#### IFAT

Immunoglobulin G antibodies were detected using *L. infantum* (strain MHOM/IT/80/IPT1) antigen slides produced by C.Re.Na.L. (Centro di Referenza Nazionale per la Leishmaniosi, Palermo, Italy). Fluoresceinated goat anti-cat immunoglobulin G (IgG) antibody (Anti-cat IgG-FITC conjugate, SIGMA, Saint Louis, Missouri, USA) diluted in PBS (from 1:180 to 1:200 according to the batch) was used. The IFAT was performed according to the manufacturer’s instructions and the end-point titer of positive samples was determined preparing serial two-fold dilutions of serum starting from 1:20. The cut-off value for positivity was established at 1:80 [5].

#### ELISA

An ELISA previously described was performed with slight modifications [11]. Briefly, each plate was coated with 100 μl/well of 20 μg/ml antigen extracted from sonicated *L. infantum* promastigote culture in 0.1 M carbonate/bicarbonate buffer (pH 9.6 at 25 °C) and incubated overnight at 4 °C. Plates were then frozen and stored at -20 °C.

One hundred microliters of cat sera, diluted 1:800 in PBS-0.05% Tween 20 (PBST)-1% dried skimmed milk (PBST-M), were added to each well and the plate was incubated for 1 h at 37 °C in moist chamber. After three washes with PBST for 3 min and one wash with PBS for 1 min, 100 μl per well of anti-cat IgG (Serotec, Bangkok, Thailand) 1:10000 in PBST-M were added and incubated for 1 h at 37 °C in moist chamber. The substrate solution (orthophenylenediamine, 0.5 mg/ml; Thermo Fisher, Waltham, Massachusetts, USA) plus H_2_O_2_ (0.4 μl/ml) in 0.1 M phosphate/citrate buffer at pH 5.0, was added at 100 μl per well and developed for 20 ± 5 min at 24 °C in the dark. The reaction was stopped with 100 μl of 2.5 M H_2_SO_4_. The optical density (OD) was measured using an automatic micro-ELISA (Anthos 2020, Cambridge, UK) at a wavelength of 492 nm.

All plates included pooled serum from three sick cats with a confirmed infection as a positive control (calibrator) and serum of a cat from an area where leishmaniosis was not endemic as a negative control and all samples were analyzed in duplicate. The reaction was quantified as ELISA units (EU) related to positive cat sera used as calibrators and arbitrarily set at 100 EU. The cut-off was established at 40 ELISA units [mean ± 4 standard deviations (SD), of sera from 87 cats from non-endemic area] [11].

#### Western blot

WB analysis was performed as described previously for the diagnosis of clinical leishmaniosis due to *L. infantum* and cutaneous leishmaniosis due to *Leishmania major* in humans [21–23]. A nitrocellulose sheet sensitized with 2 mg/ml of antigen extract from *L. infantum* promastigote culture (zymodeme, MON-1) was carried out as described [21]. The homemade nitrocellulose paper was rehydrated with 500 μl of non-fat dried milk and incubated for 30 min in slow agitation. The liquid of each gutter was removed and more 500 μl of milk were added with 40 μl of feline serum samples and only 10 μl of serum for the positive control. The bowl was left in slow agitation overnight with a lid.

After 3 washes of 5 min with solution buffer (1/10 dilution of buffer + surfactant + NaN3), 1.2 ml of conjugate anti-human [buffer + polyclonal rabbit anti-human IgG conjugated with alkaline phosphatase + NaN3 (below 0.1%) + stabilizers, LDBIO] was distributed on each gutter, the bowl was covered with a lid and incubated 1 h 30 min in slow agitation. After repeating the washes, 1.2 ml of substrate (buffer + NBT+ BCIP+ stabilizers, LDBIO) was put in each gutter and incubated with a lid in slow agitation for 20–30 min. The reaction was stopped with distilled water when the characteristic bars appeared on the positive control sample.

In WB analysis for the diagnosis of feline leishmaniosis only bands with low molecular weight (14, 18, 21, 23 and 31 kDa) were considered diagnostic [11, 24]. In particular, only the presence of the 18 kDa band was suggestive of *L. infantum* infection as described previously in cats [17, 25] and humans [22, 23].

### Statistical analysis

On each sample unit composed by a single feline serum sample tested by IFAT, ELISA and WB, statistical analysis was performed through a written routine [20] with MATLAB software using the Bayesian approach. The Bayesian approach considers uncertainties associated with all unknown quantities whether they are observed or unobserved. Inference is drawn by constructing the joint probability distribution of all unobserved quantities based on all that is known about them. Let D denote the infection status of the cat and let Y_*i*_, i = 1, 2, 3, be dichotomous test variables assuming *y*_*i*_ = {0, 1} respectively, for negative and positive results. The Se and Sp of the i-th test are *Se*_*i*_ = *P*(*y*_*i*_ = 1|*D* = 1) and *Sp*_*i*_ = *P*(*y*_*i*_ = 0|*D* = 0), respectively. We assume that the test outcomes for a given cat are independent, conditional on infection status of the cat. With three tests, for each one of the 2^3^ possible realizations is computed the joint probability:

1 $$ P\left({y}_1,{y}_2,{y}_3\right)=\pi \prod_{i=1}^3\  P s e\left({y}_i\right)+\left(1-\pi \right)\prod_{i=1}^3\  P s p\left({y}_i\right), $$

with $$ P s e\left({y}_i\right)=\left\{\begin{array}{c}\hfill 1- S{e}_i\  if\ {y}_i=0\hfill \\ {}\hfill S{e}_i\  if\ {y}_i=1\hfill \end{array}\right. $$ and $$ P s p\left({y}_i\right)=\left\{\begin{array}{c}\hfill S{p}_i\  if\ {y}_i=0\hfill \\ {}\hfill 1- S{p}_i\  if\ {y}_i=1\hfill \end{array}\right. $$

The observed number of test results in each of the eight cells in the 2 × 2 × 2 contingency table can be thought as the sum of those that are truly infected and those that are truly non-infected. Let us indicate as *d*_111_|*y*_111_ the unknown frequency of truly infected cats given the test response pattern (*y*_1_ = 1, *y*_2_ = 1, *y*_3_ = 1). It is binomially distributed (*y*_111_, *p*_111_) where *p*_111_ is the positive predictive value of test pattern *y*_111_. Using Bayes’ theorem:

2 $$ {p}_{111}= \Pr\ o b\left( D=1\left|{y}_1=1,{y}_2=1,{y}_3=1\right.\right)=\frac{\pi \prod_{i=1}^3 S{e}_i}{\pi \prod_{i=1}^3 S{e}_i+\left(1-\pi \right)\prod_{i=1}^3\left(1- S{e}_i\right)} $$

and *d*_111_ is the expectation of *d*_111_|*y*_111_. The other probabilities are similarly computed.

The latent data and observations from the joint posterior are simulated in the Bayesian approach by an iterative MCMC technique using the Gibbs sampler.

The Gibbs sampling proceeds iteratively with two steps as follows: (i) arbitrary starting values for the parameters (one prevalence, three sensitivities and three specificities) are chosen; and (ii) parameter values are substituted within binomial distributions and ${d}_{i_1{i}_2{i}_3}$ are sampled from the respective binomial distributions.

For each one of the seven parameters, the augmented beta posterior is computed. For example, for the first test, *Se*_1_ it is $beta\left({\alpha}_{s{ e}_1}+{d}_{1\dots },{\beta}_{s{ e}_1}+{d}_{0\dots}\right)$ where ${\alpha}_{S{ e}_1}$ and ${\beta}_{S{ e}_1}$ are the parameters of the beta prior of the Se and for *Sp*_1_ it is $beta\left({\alpha}_{s{ p}_1}+{y}_{0\dots }-{d}_{0\dots },\beta s{e}_1+{y}_{1\dots }-{d}_{1\dots}\right)$. The joint posterior distribution of the parameters is obtained as the product of these seven independent beta posteriors. Once the posterior distributions of all parameters approach equilibrium condition the following qth quantiles as parameters for the diagnostic test are considered: q = 0.50 for calculating the median and q = 0.025 and 0.975 for calculating the 95% credibility intervals of the sample generated from the posterior distribution. The non-informative beta prior distribution for parameters (i.e. α = 1 β = 1) of diagnostic test and prevalence sample is employed.

In this study, we used J cycles of the Gibbs sampler and the last N generations of the chain forms the sample of the equilibrium distribution [20]. The first J-N iterations ensures the convergence of all the full conditional distributions. Thus we used a vector of size N to estimate the posterior median and 95% credibility intervals of the prevalence and the Se and Sp of each test. We fix *N* = 2,000 whereas the value of J (< 3,300) depends on the rate of the convergence. We use starting values for the prevalence 0.1, for Se and Sp for each of three test respectively 0.50 and 0.80.

The routine was applied to each group of cats to evaluate the performance of the three tests in each group and in the total sample composed by 161 cats. In details, the statistical analysis at first compared simultaneously the three tests in the three groups studied and gained the true prevalence in each group. In this way it was possible to understand which test was best according to the purpose of diagnosis. Furthermore, this analysis revealed the critical points related to the estimates when sample size is small and they are not representative of the population under investigation. Then, in order to improve estimates and find the most accurate test we considered the total sample composed by the three groups of cats.

## Results

Table 1 shows the combination of results obtained by IFAT, ELISA and WB in each group of cats studied. The results of the performed simulation of Se, Sp and prevalence in the three groups is shown in Table 2.

**Table 1:** Combination of results of the three serological tests detected in each group of cats

Serological technique			Non-endemic area (*n* = 64)	Endemic area (*n* = 76)	Affected by leishmaniosis (*n* = 21)
IFAT	ELISA	WB	Number of observations		
–	–	–	61	53	0
–	–	+	2	9	0
–	+	–	1	0	0
–	+	+	0	1	1
+	–	–	0	9	0
+	–	+	0	3	0
+	+	–	0	0	0
+	+	+	0	1	20

+, positive test result; −, negative test result

**Table 2:** Output parameters of the accuracy of tests for each group of cats studied

	IFAT		ELISA		WB	
	Median	CI	Median	CI	Median	CI
Non-endemic area: sample prevalence 0.02% (CI: 0.00–0.16)						
Sensitivity	0.33	0.01–0.96	0.38	0.02–0.97	0.41	0.02–0.96
Specificity	0.99	0.94–1.00	0.98	0.92–1.00	0.96	0.90–0.99
Endemic area: sample prevalence 0.10% (CI: 0.01–0.96)						
Sensitivity	0.43	0.07–0.93	0.26	0.00–0.94	0.64	0.07–0.99
Specificity	0.84	0.18–0.95	0.98	0.30–1.00	0.84	0.06–0.96
Affected by leishmaniosis: sample prevalence 0.89% (CI: 0.01–1.00)						
Sensitivity	0.89	0.08–0.99	0.94	0.13–1.00	0.94	0.09–1.00
Specificity	0.21	0.01–0.95	0.18	0.00–0.93	0.16	0.00–0.94

*Abbreviation*: *CI* 0.95 credible interval

In cats from non-endemic area, Se of the tests was not high with best value (41%) offered by WB but with a high statistical variability (low precision) as represented by 95% credible intervals (CI) (CI: 2–96%). Obviously, the high uncertainty is determined by 95% of negative results obtained by diagnostic tests in this group and by sample size. Conversely, Sp of tests is high and with high precision and IFAT offered the best value (99%) as well as high precision (CI: 94–100%). The true prevalence calculated simultaneously by the three tests in the non-endemic area group was 2%.

More false positive and false negative results emerged in the group of cats from the endemic area. In fact the best Se (64%) and Sp (98%) respectively, given by WB and ELISA were both obtained with a low precision. Precision was low (CI: 1–96%) also for the prevalence sample (10%).

In the group of cats affected by leishmaniosis, ELISA and WB had the same estimate of Se (94%) but ELISA (CI: 13–100%) had a smaller uncertainty than WB (CI: 9–100%). As expected, in the group of sick individuals, Sp values were low and had a low precision with all tests. However, the best Sp (21%; CI: 1–95%) was obtained by IFAT. The prevalence sample in this group was 89% but with a low precision (CI: 1–100%).

One hundred and thirty-five serum sample units out of 161 (83.8%) produced results in agreement. Table *3* shows the test results for the number of cats which have one of the 8 different test patterns.

**Table 3:** Results of three serological tests applied to all 161 cats

IFAT	ELISA	WB	No. of observations
–	–	–	114
–	–	+	11
–	+	–	1
–	+	+	2
+	–	–	9
+	–	+	3
+	+	-	0
+	+	+	21

+, positive test result; −, negative test result

The results of simulation of Se and Sp of IFAT, ELISA and WB and sample prevalence in the overall 161 samples are shown in Table 4. As the sample size is larger, test accuracy was higher and provided a greater precision. Western blot was the most accurate test: Se = 97% (CI: 86–100%); Sp = 99% (CI: 96–100%). Sensitivity of IFAT (75%) was higher and with a higher precision (CI: 61–87%) compared to that of ELISA (70%; CI: 56–83%). Conversely ELISA Sp (98%) and precision (CI: 94–100%) slightly exceeded that of IFAT (97%; CI: 93–100%).

**Table 4:** Output parameters of the accuracy of the tests with all cats studied. Sample prevalence 0.27; CI = 0.20–0.34

	IFAT		ELISA		WB	
	Median	CI	Median	CI	Median	CI
Sensitivity	0.75	0.61–0.87	0.70	0.56–0.83	0.97	0.86–1.00
Specificity	0.97	0.93–0.99	0.98	0.94–1.00	0.99	0.96–1.00

*Abbreviation*: *CI* 0.95 credible interval

## Discussion

This is the first study that assesses the diagnostic performance of IFAT, ELISA and WB for the detection of anti-*L. infantum* antibodies in sera of cats from non-endemic and endemic areas (included cats with confirmed clinical leishmaniosis) using a Bayesian approach in the absence of a gold standard. Parasite specific antibody detection is a fundamental diagnostic tool for confirming a clinical suspicion of leishmaniosis in dogs [6] but the possibility of discrepancy between different serological techniques is well known in both dogs and cats [4, 9, 11, 13, 26]. Serological methods are however less useful for assessing the infection seroprevalence of dogs living in endemic areas as subclinical infections are usually associated with negative or border-line results [6]. In this study, cats with clinical leishmanosis were 100% positive with WB or ELISA and 95% with IFAT, supporting that antibody detection can be used for confirming feline leishmaniosis in clinical practice as for canine leishmaniosis. However, some caution is needed before excluding leishmaniosis in IFAT negative cats. Sick cats with clinical picture compatible with leishmaniosis but negative by IFAT should perform other serological tests or complementary diagnostic tools such as cytology, histology or PCR [1].

In endemic areas for *Trypanosoma* spp. or other *Leishmania* spp. cross reactions with *L. infantum* must be taken into account for interpretation of serological tests but this is not the case of the geographical area of investigation of this study [27–29].

The percentage of antibody positive samples to at least one technique in the group of 76 cats living in South Italy reached 30% confirming that feline *Leishmania* infection is frequent in endemic areas (2). This feline group is representative of an adult feline population admitted for heterogeneous reasons to clinical practice in endemic area. These cats are potentially exposed to the parasite and can stand in one of the following conditions: not come into contact with *Leishmania*, come into contact but not become infected, otherwise they are infected. Moreover, infected individuals can stand at the time of sampling in a different point of the wide spectrum of clinical situations going from a subclinical infection to overt disease. ELISA testing appears to have a low Se for detecting antibodies compared to WB and IFAT in subclinical infections. This finding is in contrast with other studies and is of clinical relevance when there is a need of testing cats in endemic areas because they are blood donors, before their exportation to a non-endemic area or because an immune-suppressive therapy has to be given [1, 30]. The very strict calculation that we used (mean value ± 4 SD of sera from non-endemic area) for ELISA cut-off setting obviously contributed to this result.

Despite the large number of published serological investigations, very few studies validated serological techniques testing for anti-*Leishmania* antibodies a consistent number of serum samples obtained from feline patients of non-endemic area [5, 11, 31, 32]. It is important to have as much extensive as possible data by testing feline populations of non-endemic regions in order to exclude the possibility of cross-reactions with other microbial agents or even with endogenous feline proteins. The current state of the art clearly rule out cross-reactivity with *Toxoplasma gondii* only, therefore we cannot exclude cross-reactions for some positive results we obtained [10, 14, 15, 32, 33]. It is important to remark that the travel and clinical history were unknown in all cats studied from non-endemic area and this can be considered a limitation of this study. However, the overall good discrimination that we obtained between the three different categories of cats confirms that we set appropriate methodologies for IFAT, ELISA and WB techniques. In particular, we confirmed that 80 is appropriate IFAT cut-off and the 18 kDa band is marker for positive WB when testing feline sera likewise it occurs in dogs and humans [5, 21–23].

Various problems may arise when comparing diagnostic performance of different tests given the absence of a gold standard diagnostic test for *Leishmania* infection. One problem concerns the influence of spectrum (stage of the disease) and selection (inclusion criteria) bias. For example, it is more difficult to suspect a disease at an early stage and the criteria for inclusion of patients to be tested in a study will be crucial. Ideally, each unit in the sample should be randomly selected so that the sample is representative of the target population. This is difficult to do in field studies and this type of error yields to misleading statistics. Therefore, it is important to be aware of the influence of spectrum and selection bias on the accuracy of diagnostic tests for *Leishmania* infection, but this must be kept in perspective. This means that to carefully assess the Se and Sp of diagnostic tests, samples of cats with confirmed leishmaniosis as well as of animals from endemic and non-endemic areas should be selected. Other important factors influencing the diagnostic performance of tests include technical variations. In this study, cut-off values used for IFAT and ELISA and the criteria for interpretation of positive immunoblots in feline sera were confirmed to be appropriate for discriminating samples from endemic and non-endemic areas and, among those from endemic area, between cats with confirmed leishmaniosis and cats with other clinical problems. The aim of this study was to evaluate the diagnostic performance of tests frequently used for the detection of anti-*Leishmania* antibodies in feline sera in the absence of a gold standard. For the first time, this analytical problem was solved by writing a routine with MATLAB software in the Bayesian framework. This approach has the main advantage of drawing inference from diagnostic tests in the absence of a gold standard. The non-informative beta prior for parameter provided a minimal effect on the final inference of three diagnostic tests and we obtained the maximum of information from the experimental data. One weak point of this study is, however, given by the sample size of cats with confirmed leishmaniosis compared to those non-infected that we could test, as this can be one possible source for uncertainty of Se of ELISA (27%) and IFAT (26%) compared to that of WB (14%). The analysis of a more extensive and better balanced specimen of feline serum samples can confirm or reject this hypothesis.

## Conclusion

The diagnostic performance of serological tests frequently used for the detection of anti-*Leishmania* antibodies in cats was evaluated in the absence of a gold standard in the Bayesian framework. The overall performance of WB, IFAT and ELISA was good, but WB targeting for positivity the 18 kDa band offered the best diagnostic performance for the detection of antibodies against *L. infantum*. Overall, IFAT (cut-off 80) was more sensitive than ELISA to detect subclinical or early infections while ELISA was better for diagnosing clinical leishmaniosis when compared with IFAT. As there is no perfect diagnostic test, the best choice for each specific purpose of diagnosis (infection *versus* disease) theoretically is offered by WB because of the highest Se and Sp, however the analysis of more samples is necessary for confirming our results.

## Abbreviations

ELISA: Enzyme-linked immunosorbent assay
IFAT: Immunofluorescence antibody test
MCMC: Markov Chain Monte Carlo
WB: Western blot
